# Chromosome Mechanics and Meiotic Engine Maintenance

**DOI:** 10.1371/journal.pgen.1000210

**Published:** 2008-09-26

**Authors:** Michael E. Dresser

**Affiliations:** Oklahoma Medical Research Foundation, Oklahoma City, Oklahoma, United States of America

The behavior of chromosomes during meiosis has been likened to a middle school dance, where partners find one another, form couples that move about and trade information, and then separate to opposite sides of the dance hall. With chromosomes, as with the dancers, forming exclusive couples often is difficult—individuals can be attracted to more than one partner or find themselves trapped behind or between other couples—and, failing to form a couple effectively, end up on the wrong side of the dance hall. For chromosomes, this failure of pairing and segregation leads to an unbalanced chromosome complement (aneuploidy), with its attendant problems of sterility and genetic disease. Two papers in this issue of *PLoS Genetics* [1],[2] demonstrate that telomere-promoted movements influence nearly every step in chromosome pairing and meiotic recombination, opening a new avenue to address questions that have intrigued biologists and vexed clinicians for over a hundred years.

**Figure 1:**
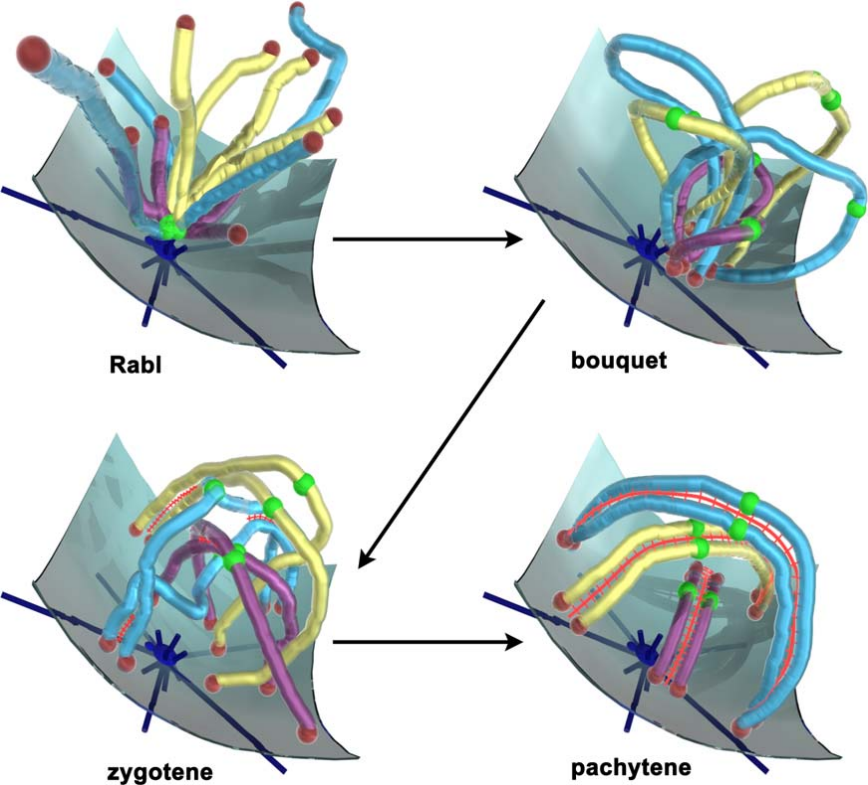
Chromosomes Pass through Distinct Organizational Phases as They Negotiate Meiotic Prophase. The Rabl orientation is established in the prior division, where centromeres (green balls) are pulled to the spindle pole (dark blue structure with emanating microtubules, anchored in the nuclear membrane), with the telomeres (red balls) trailing. Early in prophase, centromeres move away from the pole while telomeres attach to the nuclear membrane and move to a small area adjacent to the spindle pole, forming the bouquet. Chromosomes move out of the bouquet as the synaptonemal complex forms (red ladder-like structure), marking entry into zygotene and generating interlocks, where chromosomes are trapped between synapsing pairs. Completion of synapsis and resolution of interlocks marks pachytene, where chromosome pairs appear well separated.

Chromosome movement is implicit in the classically recognized stages of meiotic prophase, but descriptions of directly visualized movements have been rare (see [3]). Early in prophase, chromosomes transition from having their centromeres clustered near the spindle pole (the Rabl orientation) to having their telomeres clustered at the nuclear periphery adjacent to the spindle pole (the bouquet orientation; see Figure 1). The bouquet stage ends with dispersal of telomeres across the inner nuclear envelope as chromosomes finalize their intimate pairing by forming synaptonemal complexes (SCs) that link chromosome pairs closely along their lengths. The formation of these intimate, exclusive partnerships would seem to finish the task at hand and to end the need for active, whole-chromosome movements, but this turns out not to be the case.

Movements that persist throughout meiotic prophase were first described by Hiraoka's group in the fission yeast *Schizosaccharomyces pombe* where, following bouquet formation, telomeres remain at the spindle pole while it leads the nucleus along microtubules, back and forth through the cell, until just before the first meiotic division [4]. Meiotic prophase is noncanonical in *S. pombe* in that synaptonemal complexes are not formed and recombination is not regulated to avoid forming crossovers near one another (i.e., there is no positive crossover interference). This has led some to question the generality of persistent movements. Recently, however, similarly persistent rapid prophase movements (RPMs) have been described in the budding yeast *Saccharomyces cerevisiae* [5]–[7]. Although these movements are of individual chromosomes rather than of the whole genomic complement, and although they appear to be promoted by actin rather than by microtubules, each system involves SUN domain–containing proteins that are known to mediate transnuclear envelope linkages, in the present case tethering telomeres to the cytoskeleton [8]–[10]. Such linkages also are present in mammalian meiotic nuclei [11],[12], indicating a widely conserved mechanism and suggesting conserved function(s).

Before its role in these movements was recognized, the budding yeast Ndj1 protein was known to promote bouquet formation, the normal kinetics of SC formation, and the usual pattern of meiotic recombination; to maintain low levels of ectopic recombination (genetic exchanges between homologous DNA sequences in nonallelic locations); and, ultimately, to reduce the frequency of aneuploidy [13]–[17]. Ndj1 also plays a role in anchoring telomeres to the inner nuclear envelope [15], apparently by stabilizing the association of telomeres with the transmembrane SUN protein, Mps3 [10]. Reports that the meiosis-specific budding yeast protein Csm4 is similar to Ndj1 in being required to prevent aneuploidy [18] led the authors of the two current papers to ask, in remarkable molecular detail, whether the meiotic requirements for Csm4 are similar to those for Mps3 and Ndj1. The simple answer is “Yes,” but the angel is in the details.

The authors find that Csm4, unlike Mps3 and Ndj1, is *not* required to anchor telomeres to the nuclear envelope but is required for telomeres to engage in the RPMs (see [7],[8]). Nevertheless, the impact on the progress of recombination, in all its currently understood molecular intricacies, is similar—delays in the appearances of recombination intermediates begin very early in prophase and persist or lengthen as prophase progresses. The implication of these observations is that the RPMs are the critical factor rather than telomere tethering to the nuclear envelope per se. So then, what is the role of the RPMs? Here, the authors diverge somewhat in their answers. The paper from the Shinohara lab proposes that RPMs promote the biochemistry of recombination more or less directly, perhaps by affecting chromosome structure [1]. The paper from the Alani and Kleckner labs proposes that RPMs function during an early phase when the cell determines which early recombination intermediates will become crossovers. They suggest that delays in this phase, perhaps due to a requirement for Ndj1 and Csm4 to resolve chromosome interlocks (at zygotene, see Figure 1), generates the subsequent defects [2]. Tests of these hypotheses will require considerable ingenuity in experimental design.

A simple and striking conclusion from these papers is that mechanical energy, pumped into the nucleus via the telomeres, contributes critically to the work of genetic recombination. Identification of the MNC complex (Mps3, Ndj1, Csm4) in *Sa. cerevisiae* and of related structures and pathways in other organisms is only the beginning to understanding how these transnuclear envelope tethers are constructed and regulated. Understanding how these connections and the movements they foster contribute to the faithful segregation of chromosomes in meiosis will be challenging and rewarding, like the middle school dance.
